# The pre-analytical process management status and influencing factors of laboratory test before prescribing antimicrobial in developing country

**DOI:** 10.1186/s12913-023-09243-8

**Published:** 2023-03-25

**Authors:** Feiyang Zheng, Kang Wang, Qianning Wang, Tiantian Yu, Xinping Zhang

**Affiliations:** 1 School of Medicine and Health Management, Tongji Medical College of Huazhong University of Science and Technology, Wuhan, 430030 Hubei China; 2 School of Nursing, Hong Kong Polytechnic University, Hung Hom, Kowloon, Hong Kong SAR

**Keywords:** Pre-analytical process, Laboratory test, Antimicrobial, Management

## Abstract

**Introduction:**

The results of laboratory testing are crucial basis for clinicians to prescribe antimicrobial. Laboratory testing is a highly complex process, and increasing evidence suggests that errors and obstacles in the pre-analytical process (PP) will affect reasonable antimicrobial use. However, PP was an easily neglected link in hospital infection management and the current situation of it and the influencing factors of management are not clear.

**Methods:**

A cross-sectional survey was conducted in the department of clinical, specimen collection, transportation, and inspection in 109 secondary and tertiary hospitals in Central China. The rate of antimicrobial susceptibility test request (AST) and related indexes of above departments were calculated to describe the situation. Management characteristics (frequency of training etc.) were described as proportions and fractional probit regression analysis was used to determine the influencing factors.

**Results:**

The average rate of non restricted-use antimicrobial was 63%, the restricted-use was 86%, the special-use was 95%. The zero obstacle rate of specimen collection was 27.3%, of specimen transportation was 19.4% and of inspection feedback was 61.7%. There was a difference between the secondary and tertiary hospitals on non restricted-use (X^2^ = 22.968, *P* < 0.001); restricted-use (X^2^ = 29.466, *P* < 0.001); special-use (X^2^ = 27.317, *P* < 0.001). Taking non restricted-use as an example, training (OR = 0.312, 95%CI: 0.148,0.429), low-frequency appraisal (OR = 0.153, 95%CI: 0.082,0.224), guidance (OR = 0.32, 95%CI: 0.237,0.403) and information technology (OR = 0.104, 95%CI: 0.009,0.199) were positive factors.

**Conclusions:**

There were substantial differences in the rate of AST request in clinical department between secondary and tertiary hospitals. The zero obstacle rate in collection, transportation and inspection department were still low. In most departments, training and performance appraisal were positive factors, guidance and information technology were positive supporting factors.

**Supplementary Information:**

The online version contains supplementary material available at 10.1186/s12913-023-09243-8.

## Introduction

Antimicrobial resistance (AMR) is one of the biggest public health and development threats [1]. Misuse and overuse of antimicrobial are the main drivers in the development of drug-resistant pathogens. At least 2.8 million people get an antibiotic-resistant infection, and more than 35,900 people die every year in the U.S. [2, 3]. The coronavirus disease (COVID-19) pandemic further demonstrates the challenge of AMR [4]. During the pandemic, a large number of biocides and disinfectants were used, resulting in the release of antibacterial agents into the environment and the selection and the development of highly resistant bacteria [5, 6]. Since there is no targeted treatment for the time being, many patients with COVID-19 received antibiotic treatment, which will cause serious compound infection [7]. Therefore, it is critical to implement effective antibiotic use management, especially in developing countries. Microbiology laboratory diagnosis is considered an important factor affecting clinicians’ prescription of antimicrobial, especially through pathogen identification and reporting the results of antibiotic sensitivity tests, which is very important to guide clinicians to use antimicrobial safely and economically [8–10]. Simões et al. showed that the level of confidence in antibiotic prescribing reached 100% when the microbiological results were known [11].

The process of laboratory test before prescribing antimicrobial can be divided into three phases (pre-, intra- and post-analytical). The pre-analytical phase (PP) involves a range of workflows in multi-department, including the test application of clinician, collection of patient specimens, transportation of specimens, and feedback of inspection department [12]. Clinicians, nurses, specimens transporters, and inspectors participate in this workflow. Errors or barriers in any part of this closed-loop will affect the microbial test results, leading to unreasonable use of antimicrobial [13, 14]. The antimicrobial stewardship program (ASP) recommended that microbiology labs and stewardship programs can work together to optimize the use of such tests and the communication of results [15]. Studies have confirmed that various management measures such as education, feedback, and reminders are effective in improving the quality of the pre-analytical phase [16]. However, at present, the management of PP mainly focuses on the improvement of experimental technology and evaluation of specimen quality [17, 18]. And the main intervention measures included training and evaluation of quality scores, which were implemented in clinical, collection and inspection departments respectively. There was a lack of systematic supervision and management for all departments and there was no comprehensive research on the management factors and levels affecting PP. While a small number of studies have confirmed the improved quality of PP through management interventions, the studies are limited to a specific hospital and the results are not generalizable [19, 20].

In the context of increasingly pursuing evidence-based medicine, useful experimental results depend on high-quality PP (involving a series of processes and collaboration with multiple departments), especially in developing regions and countries with uneven distribution of medical resources [13, 21]. Chinese government has proposed to improve the request rate of AST before antimicrobial treatment as an index for medical quality improvement in 2021 and defined three using types of antimicrobial, in which the request rate of non-restricted use antimicrobial should not be less than 30%, the restricted-use antimicrobial should not be less than 50%, and the special-use antimicrobial should not be less than 80% [22]. Taking cephalosporins as an example, the non-restricted antimicrobial include cephalexin and ceftriaxone, the restricted antimicrobial include cefprozil, cefotaxime, etc., and the special-use antimicrobial include cefotaxil, ceftriaxone, etc. Luo ester and so on. Moreover, the management of the request of microbiological inspection was included in the scope of ASP. Based on this, this study conducted a cross-sectional survey of the operational and supervisory status of PP in various departments in Central China from the perspective of managers, investigate the management related factors affecting the development of PP, and identify priority areas to improve ASP, providing a reference scheme for other developing countries and areas with limited medical resources.

## Materials and methods

### Study design and participants

This cross-sectional survey was a self-administered questionnaire distributed to all secondary and tertiary hospital departments related to PP (including collection department, specimen transport department, inspection department) and several clinical departments with a high frequency of antimicrobial use (respiratory medicine, urology, ICU, neurology, endocrinology, and orthopedics, etc.) in Hubei, China. The area where the questionnaire was sent in this study is consistent with the distribution of medical and health resources in Hubei Province. On the whole, the six cities of Wuhan, Huangshi, Shiyan, Yichang, Enshi and Xiangyang are the areas with the highest amount of medical and health resources. And the facilities we surveyed covered these areas. This survey was implemented in June 2, 2021 and the questionnaires were recalled after 1 week with a reminder. According to the degree of participation in the pre-analysis process and the ratio of human resources of each hospital, one director of the infection control department, at least two staff of the transportation department and the inspection department, at least five clinicians of key departments and eight nurses of each hospital were required to fill in the questionnaire. Relying on the Hubei Provincial Infection Management and Control Center, we distributed questionnaires to all hospitals under its jurisdiction, with a response rate of 100%. After screening, 109 hospitals’ responses met the quality requirements of this study and were included in the analysis. There are 109 infection control managers, 2539 clinicians, 4215 nurses, 980 specimen transporters and 266 specimen inspectors.

### Questionnaire and indicators

To prepare the questionnaire, this study reviewed relevant articles on the practice and impact assessment of antimicrobial management plans at both the institutional and individual levels. Based on *Specimen collection and transport in clinical microbiology* by the National Health Commission of the People’s Republic of China in 2018 and the *2015 edition of the Guiding Principles of Clinical Application of antibiotic*, the issues of supervision are designed from the perspective of management or policy implementation. And then follow-ups with the evaluation by expert judges or panels. Experts involved in the study included five hospital infection prevention and control staff, five clinicians and nurses in key departments, and five relevant personnel involved in the inspection. We used the content validity ratio (CVR) to evaluate the content validity of each item in the questionnaire. Refer to the minimum acceptable value of the content validity ratio reported by Lawshe, the evaluation results of 15 experts are greater than the acceptable minimum value of 0.49, indicating that the content validity of this questionnaire is good. Further details of the research design can be obtained at the hospital infection center of Hubei Province (whcdc. ORG). Eventually, the questionnaire was divided into five parts by different survey departments and subjects.

Almost every part involved the investigation of demographic characteristics, the status of regulatory systems related to PP, performance appraisal, publicity and education, etc. In addition, we designed management evaluation indicators for very department (request rate of non-restricted use of antimicrobial (NR), request rate of restricted use of antimicrobial (RR), request rate of special-grade use of antimicrobial (SR), zero obstacle rate of specimen collection (ZR), zero obstacle rate of specimen transportation (TR), zero obstacle rate of specimen inspection feedback (FR)) to reflect the situation of hospital supervision of PP. The specific definitions and calculation formulas were displayed in Table 1. These indicators were designed to evaluate the management impact under the best conditions of PP.

**Table 1:** The supervision indicators of hospital for inspection examination

Index	Definition	Calculation formula
Request rate of non restricted antimicrobial (NR)	The proportion of clinicians requesting microbiological laboratory diagnostic tests before prescribing non restricted antimicrobial	The number of clinicians requesting microbiological laboratory diagnostic tests before prescribing non restricted antimicrobial / total number of clinicians
Request rate of restricted antimicrobial (RR)	The proportion of clinicians requesting microbiological laboratory diagnostic tests before prescribing restricted antimicrobial	The number of clinicians requesting microbiological laboratory diagnostic test before prescribing restricted antimicrobial / total number of clinicians
Request rate of special-use antimicrobial (SR)	The proportion of clinicians requesting microbiological laboratory diagnostic tests before prescribing special-use antimicrobial	The number of doctors requesting microbiological laboratory diagnostic test before prescribing special-use antimicrobial / total number of clinicians
Zero obstacle rate of specimen collection (ZR)	The proportion of nurses who did not encounter obstacles mentioned in the questionnaire during sample collection	The number of nurses / total number of nurses who did not encounter the collection obstacles mentioned in the questionnaire
Zero obstacle rate of specimen transportation (TR)	The proportion of persons who did not encounter obstacles mentioned in the questionnaire during sample delivery	The number of transporters who did not encounter the transportation obstacles mentioned in the questionnaire/total number of transporters
Zero obstacle rate of specimen inspection feedback (FR)	The proportion of clinical laboratory staff who feed back unqualified samples to clinicians/ nurses.	The number of clinical laboratory staff giving feedback / total number of clinical laboratory inspectors

### Survey administration

Questionnaire was sent to the infection control department of each hospital by e-mail. The person in charge distributed it to each part, collected it after 1 week and conducted quality audits, then returned it by email in a specific format. The questionnaire was coded according to medical institutions for summary and sorting. EpiData is used for data entry of paper questionnaire. In order to reduce the bias in the investigation process, we have carried out the following work: 1) Firstly, we held a meeting with each IPC manager before investigation. We fully informed them of the survey objectives and specific content at the meeting and informed them of the specific requirements for distribution, collection and audit of questionnaires. 2) We created a review section on questionnaire requiring the person in charge to check and sign when the questionnaire is received. 3) Our research team arranged specially-assigned persons to liaise with each hospital and the problems faced by IPC managers can be resolved in a timely manner. If necessary, we will go offline for guidance. 4) During the investigation, the IPC managers sent the recycled questionnaire to us via email in the form of electronic scanning every day. Team members will further audit to ensure that the respondents meet the requirements.

### Statistics analysis

Descriptive statistical analysis is used to analyze the different distribution of hospital department characteristics and management factors. Fractional probit regression model was applied to analyze the influencing factors because the values of dependent variables were between zero and one and may also be equal to zero or one. Fractional probit regression model is an extension of generalized linear model (GLM) [23]. It is a kind of functional forms, which avoids most known issues of traditional econometric models for bounded variables [24]. Fractional response estimators fit models on continuous zero to one data using probit, logit, heteroskedastic probit, and beta regression. Probit regression can be used when the endpoints zero and one are included. The log-likelihood function for fractional models is of the form:

$$\ln L=\sum_{j=1}^Nw_jy_j\ln\left\{\phi\left(X'_j\beta\right)\right\}+w_j\left(1-y_j\right)\ln\left\{1-\phi\left(X'_j\beta\right)\right\}$$

where N is the sample size, yj is the indicator, wj denotes the optional weights, lnL is maximiz, where xj are the covariates for individual j, zj are the covariates used to model the variance of the outcome for the heteroskedastic probit model, and Φ is the standard normal cumulative density function.

## Result

### Characteristics of respondents and the management status of PP

Among the 109 hospitals surveyed, there are 60 secondary hospitals and 49 tertiary hospitals. The hospitals that responded covered the area with the highest amount of medical and health resources, such as Wuhan, Huangshi, Shiyan, Yichang, Enshi and Xiangyang and so on. The demographic characteristics of each department were shown in Table 2. PP related management team members have a single professional background, mostly nursing (88.1%). More than half of the respondents from the clinical department (secondary hospital: 63.4%; tertiary hospital: 60.8%) and the collection department (secondary hospital: 63.4%; tertiary hospital: 60.8%) claimed that the performance appraisal frequency was once a month, and nearly half of the respondents from the inspection department (secondary hospital: 46.7%; tertiary hospital: 44.2%) said that there was no appraisal. However, there was no appraisal related to PP in the transportation department. For training and publicity, the proportion of respondents who participated once a year in all departments was higher. More than 90% of the collection and inspection departments were supported by related guidelines. Information systems were used to support inspection departments (93.2%) more than clinical (85.6%) and collection department (81.7%) (Table 3).

**Table 2:** Characteristics of hospital departments and different distribution of management factors

Department type	Characteristics	Total	Hospital distribution	
			Secondary hospital	Tertiary hospital
Clinical department		2539		
	Gender (female)	813 (32%)	404 (15.9%)	409 (16.1%)
	Age (mean)		36	37
	Professional Medical Title			
	Senior and senior associate	590 (23.2%)	279 (11%)	311 (12.2%)
	Intermediate	1135 (44.7%)	565 (22.3%)	570 (22.4%)
	Primary and below	814 (32.1%)	480 (18.9%)	334 (13.2%)
Collection department		4215		
	Gender (female)	4134 (98.1%)	2204 (52.3%)	1930 (45.8%)
	Age (mean)		31	31
	Professional Medical Title			
	Senior and senior associate	115 (2.7%)	41 (1%)	74 (1.8%)
	Intermediate	1243 (29.5%)	602 (14.3%)	641 (15.2%)
	Primary and below	2857 (67.8%)	1587 (37.7%)	1270 (30.1%)
Transport department		980		
	Gender (female)	900 (91.8%)	498 (50.8%)	402 (41%)
	Age (mean)		33	34
	Professional Medical Title			
	Senior and senior associate	49 (5%)	22 (2.2%)	27 (2.8%)
	Intermediate	257 (26.2%)	158 (16.1%)	99 (10.1%)
	Primary and below	674 (68.8%)	358 (36.5%)	316 (32.2%)
Inspection department		266		
	Gender (female)	173 (65%)	95 (35.7%)	78 (29.3%)
	Age (mean)		37	40
	Professional Medical Title			
	Senior and senior associate	63 (23.7%)	22 (8.3%)	41 (15.4%)
	Intermediate	121 (45.5%)	55 (20.7%)	66 (24.8%)
	Primary and below	82 (30.8%)	60 (22.6%)	22 (8.3%)
Infection management department		109		
	Gender (female)	99 (90.8%)	58 (53.2%)	41 (37.6%)
	Age (mean)		46	46
	Professional background			
	Nursing	96 (88.1%)	58 (53.2%)	38 (34.9%)
	Clinical medicine	9 (8.3%)	–	9(%)
	Public Health	4 (3.7%)	2 (1.8%)	2 (1.8%)
	Education			
	Bachelor degree or above	85 (78%)	42 (38.5%)	43 (39.4%)
	Below bachelor degree	24 (22%)	18 (16.5%)	6 (5.5%)
	Professional Medical Title			
	Senior and senior associate	71 (65.1%)	35 (32.1%)	36 (33%)
	Intermediate	28 (25.7%)	17 (15.6%)	11 (10.1%)
	Primary and below	10 (9.2%)	8 (7.3%)	2 (1.8%)

**Table 3:** Distribution of management factors and support of various departments in different hospitals on PP

Management and support type of of various departments	Total	Secondary hospital (%)					Tertiary hospital (%)				
**Performance appraisal**		None	Once a month	2 times / month	3 times / month	4 times / month	None	Once a month	2 times / month	3 times / month	4 times / month
Clinicians	2539	328 (24.8%)	839 (63.4%)	64 (4.8%)	40 (3%)	53 (4%)	282 (23.2.%)	739 (60.8%)	85 (7%)	41 (3.4%)	68 (5.6%)
Collection nurses	4215	805 (36.1%)	1274 (57.1%)	56 (2.5%)	47 (2.1%	48 (2.2%)	625 (31.5%)	1097 (55.3%)	71 (3.6%)	76 (3.8%)	116 (5.8%)
Specimen transporters		–					–				
Inspectors	266	64 (46.7%)	66 (48.2%)	1 (0.7%)	4 (3%)	2 (1.5%)	57 (44.2%)	60 (46.5%)	6 (4.7%)	3 (2.3%)	3 (2.3%)
**Training (frequency)**		None	Once a year	2 times / year	3 times / year	4 times / year	None	Once a year	2 times / year	3 times / year	4 times / year
Clinicians	2539	81 (6.1%)	521 (39.4%)	382 (28.9%)	154 (11.6%)	186 (14%)	53 (4.4%)	442 (36.4%)	400 (32.9%)	119 (9.8%)	201 (16.5%)
Collection nurses	4215	420 (18.8%)	1394 (62.5%)	147 (6.6%)	121 (5.4%)	148 (6.6%)	380 (19.1%)	1233 (62.1%)	83 (4.2%)	140 (7.1%)	149 (7.5%)
Specimen transporters	980	60 (11.2%)	275 (51.1%)	75 (13.9%)	22 (4.1%)	106 (19.7%)	65 (14.7%)	196 (44.3%)	88 (19.9%)	24 (5.4%)	69 (15.6%)
Inspectors	266	10 (7.3%)	78 (56.9%)	33 (24.1%)	8 (5.8%)	8 (5.8%)	7 (5.4%)	53 (41.1%)	47 (36.4%)	2 (1.6%)	20 (15.5%)
**Publicity (frequency)**		None	Once a year	2 times / year	3 times / year	4 times / year	None	Once a year	2 times / year	3 times / year	4 times / year
Clinicians	2539	130 (9.8%)	464 (35%)	345 (26.1%)	140 (10.6%)	245 (18.5%)	72 (5.9%)	401 (33%)	351 (28.9%)	129 (6.5%)	262 (21.6%)
Collection nurses	4215	648 (29.1%)	1175 (52.7%)	141 (6.3%)	132 (5.9%)	134 (6%)	602 (30.3%)	1018 (51.3%)	84 (4.2%)	99 (5%)	182 (9.2%)
Specimen transporters	980	98 (18.2%)	237 (44.1%)	85 (13.9%)	11 (2%)	107 (19.9%)	111 (25.1%)	172 (38.9%)	57 (12.9%)	31 (7%)	71 (16.1%)
Inspectors	266	18 (13.1%)	63 (46%)	31 (24.1%)	5 (3.6%)	20 (14.6%)	8 (6.2%)	57 (44.2%)	38 (29.5%)	8 (6%)	18 (14%)
**supervision system / guidelines (yes)**											
Clinicians	2202 (86.7%)	1137 (51.6%)					1065 (48.4%)				
Collection nurses	3813 (90.5%)	1966 (51.6%)					1847 (48.4%)				
Specimen transporters	823 (84%)	482 (58.6%)					341 (41.4%)				
Inspectors	259 (97.4%)	132 (51%)					127 (49%)				
**Informatization (yes)**											
Clinicians	2173 (85.6%)	1083 (49.8%)					1090 (51.2%)				
Collection nurses	3445 (81.7%)	1893 (54.9%)					1552 (45.1%)				
Specimen transporters	–	–					–				
Inspectors	248 (93.2%)	130 (52.4%)					118 (47.6%)				

There was a significant difference in the PP before prescribing antimicrobial of clinicians in secondary and tertiary hospital before the use of non-restricted use (X^2^ = 22.968; *P* < 0.001), restricted use (X^2^ = 29.466; *P* < 0.001) and special-grade use antimicrobial (X^2^ = 27.317; *P* < 0.001). The proportion of that in tertiary hospitals was higher when prescribing non-restricted antimicrobial, while that in secondary hospitals was higher when prescribing restricted and special-grade antimicrobial (Table 4). And there was a significant difference also in the collection (X^2^ = 41.256; *P* < 0.001), transportation (X^2^ = 29.835; *P* < 0.001) and inspection (X^2^ = 6.514; *P* = 0.038) departments between the two levels of hospital. The rate of zero obstacle of collection was 27.3% and of transportation was 19.4%. Nurses in the collection department mainly encountered obstacles such as bacterial infection at the collection site (34.2%) and insufficient sample collection volume (39.3%). In the process of transporting samples, too many samples (51.3%) and long delivery time (30.4%) were the interference obstacles with more feedback from the transportation staff. For inspectors, lack of timely feedback channels was the mainly obstacles (38.3%) (Table 5).

**Table 4:** Clinicians’ request of hospitals at different levels of antimicrobial etiology (*N* = 2539)

Request for inspection	Secondary hospital (%)	Tertiary hospital (%)	Total (%)	X^**2**^/P
Non-restricted use antimicrobial				
Yes	774 (48.7%)	814 (51.3%)	1588 (62.5%)	22.968 *P* < 0.001
No	147 (53.2%)	129 (46.7%)	276 (10.9%)	
Not sure	403 (59.7%)	272 (40.3%)	675 (26.6%)	
Restricted-use antimicrobial				
Yes	1101 (50.2%)	1092 (49.8%)	2193 (86.4%)	29.466 *P* < 0.001
No	72 (74.2%)	25 (25.8%%)	97 (3.8%)	
Not sure	151 (60.6%)	98 (39.4%)	249 (9.8%)	
Special-use antimicrobial				
Yes	1227 (51.1%)	1172 (48.9%)	2399 (94.5%)	27.317 *P* < 0.001
No	55 (83.3%)	11 (16.7%)	66 (2.6%)	
Not sure	42 (56.8%)	32 (43.2%)	74 (2.9%)

**Table 5:** Obstacles encountered during collection and transportation and inspection

Obstacles		Hospital type		Total (%)	X^**2**^/P
**During collection**	***N*** **= 4215**	**Secondary hospital**	**Tertiary hospital**		
Bacterial pollution		793 (55%)	650 (45%)	1443 (34.2%)	41.256 *P* < 0.001
Incorrect collection position		367 (53.5%)	319 (46.5%)	686 (16.3%)	
Lack of appropriate technology		393 (59.4%)	269 (40.6%)	662 (15.7%)	
Insufficient sample collection		908 (54.9%)	747 (45.1%)	1655 (39.3%)	
Without marking with relevant information		255 (60.6%)	166 (39.4%)	421 (1%)	
Lack of suitable sample containers		310 (44.5%)	387 (55.5%)	697 (16.5%)	
With out above obstacles (Zero obstacle)		553 (48%)	598 (52%)	1151 (27.3%)	
**During transportation**	***N*** **= 980**				
Too many samples to send for inspection		252 (50.1%)	251 (49.9%)	503 (51.3%)	29.835 *P* < 0.001
Without suitable transport containers		116 (59.8%)	78 (40.2%)	194 (19.8%)	
Long delivery time		113 (37.9%)	185 (62.1%)	298 (30.4%)	
Nonstandard packaging and marking		90 (55.2%)	73 (44.8%)	163 (16.6%)	
Failure to handle sample in time		132 (55.9%)	104 (44.1%)	236 (24.1%)	
Without above obstacles (Zero obstacle)		116 (61%)	74 (39%)	190 (19.4%)	
**During inspection**	***N*** **= 266**				
Lack of timely feedback channels		69	33	102 (38.3%)	6.5137 *P* = 0.038
Communication fails to reach consensus		49	20	69 (26%)	
No regular communication		64	12	76 (28.6%)	
Without above obstacles (Zero obstacle)		68	96	164 (61.7%)	

### Management factors affecting PP of each department

According to the analysis of the fractional probit regression model, we found that for all departments, the provision of guidance and training had a significant positive impact on PP. Low frequency performance appraisals had a positive impact on clinical departments and collection departments. However, the publicity of PP had no significant positive impact on most indicators.

Specifically, for the PP of three types of antimicrobial, training had a positive and significant impact on it. The monthly performance appraisals frequency also had a positive impact, and only high-frequency appraisal had a positive impact on the request rate of restricted and special-use antimicrobial. In addition, information system support had a significant positive impact on the inspection of non-restricted and restricted types of antimicrobial. It can be explained that clinicians’ interpretation of the test results before the use of restricted and non restricted antimicrobial needs more information technology based auxiliary support.

For the collection department, providing guidance and training had a positive impact on nurses’ zero barrier of collection, and compared with low-frequency assessment had a positive impact. Publicity twice a year also had a positive impact, while the frequency of four or more times a year had a negative impact. It can be found that low-frequency performance appraisal and publicity helped to promote zero obstacles in the collection process. For the transportation department, training had a positive impact on the zero interference rate, while compared with no publicity, three times a year had a negative impact on that. For the laboratory department, performance appraisal, training and publicity all had a significant positive impact on promoting the problem feedback and communication between the inspectors and the clinical department and the collection department (Table 6).

**Table 6:** Management factors influencing request of inspection: results of the fractional probit regression analysis

Departments	NR (OR-95%CI)	RR (OR-95%CI)	SR (OR-95%CI)	ZR (OR-95%CI)	TR (OR-95%CI)	FR (OR-95%CI)
**Clinical departments**						
**Training (frequency)**						
Once a year	0.312^a^	0.374^a^	0.579^a^			
	(0.148,0.429)	(0.199,0.548)	(0.407,0.751)			
2 times / year	0.399^a^	0.543^a^	0.69^a^			
	(0.229，0.568)	(0.358,0.726)	(0.504,0.876)			
3 times / year	0.391^a^	0.578^a^	0.813^a^			
	(0.209，0.573)	(0.375,0.781)	(0.603,1.022)			
4 times or more / year	0.246^a^	0.454^a^	0.515^a^			
	(0.064，0.429)	(0.265,0.643)	(0.313,0,717)			
**Publicity (frequency)**						
Once a year	−0.076	−0.173^a^	−0.041			
	(−0.212,0.06)	(−0.318,-0.027)	(− 0.185,0.104)			
2 times / year	−0.129	− 0.311^a^	− 0.151			
	(− 0.275,0.018)	(− 0.48,-0.142)	(−0.32,0.017)			
3 times / year	0.15	−0.308^a^	0.08			
	(−0.313,0.127)	(−0.488,-0.128)	(− 0.281,0.122)			
4 times or more / year	0.051	−0.109	0.107			
	(−0.101,0.204)	(−0.273,0.055)	(− 0.091,0.305)			
**Performance appraisal (frequency)**						
Once a month	0.153^a^	0.218^a^	0.192^a^			
	(0.082,0.224)	(0.125,0.31)	(0.09,0.294)			
2 times / month	0.01	0.206^a^	0.145			
	(−0.017,0.213)	(0.069,0.343)	(− 0.008,0.299)			
3 times / month	0.1	0.143	0.032			
	(−0.05,0.241)	(−0.041,0.327)	(− 0.148,0.212)			
4 times or more / month	−0.084	0.171^a^	0.179^a^			
	(−0.22,0.052)	(0.05,0.292)	(0.017,0.34)			
guidance	0.32^a^	0.069	0.238^a^			
	(0.237,0.403)	(−0.029,0.166)	(0.124,0.352)			
Informatization	0.104^a^	0.355^a^	0.281^a^			
	(0.009,0.199)	(0.257,0.453)	(0.159,0.403)			
**Collection department**						
guidance				0.246^a^		
				(0.15,0.342)		
**Performance appraisal**						
Once a month				0.187^a^		
				(0.123,0.25)		
2 times / month				−0.22^a^		
				(−0.354,-0.086)		
3 times / month				−0.063		
				(−0.242,0.116)		
4 times or more / month				−0.059		
				(−0.195,0.078)		
**Training (frequency)**						
Once a year				0.237^a^		
				(0.159,0.315)		
2 times / year				0.272^a^		
				(0.134,0.411)		
3 times / year				0.264^a^		
				(0.118,0.410)		
4 times or more / year				0.573^a^		
				(0.431,0.715)		
**Publicity (frequency)**						
Once a year				0.016		
				(−0.0451,0.083)		
2 times / year				0.147^a^		
				(0.015,0.279)		
3 times / year				0.047		
				(−0.087,0.18)		
4 times or more / year				−0.15^a^		
				(−0.274,-0.027)		
**Transportation Department**						
guidance					0.022	
					(−0.138,0.183)	
**Training (frequency)**						
Once a year					0.311^a^	
					(0.094,0.529)	
2 times / year					0.187	
					(−0.094,0.468)	
3 times / year					0.654^a^	
					(0.239,1.069)	
4 times or more / year					1.382^a^	
					(0.972,1.791)	
**Publicity (frequency)**						
Once a year					0.067	
					(−0.107,0.241)	
2 times / year					0.090	
					(− 0.166,0.345)	
3 times / year					−0.624^a^	
					(−1.051,-0.197)	
4 times or more / year					−0.364	
					(− 0.734,0.006)	
**Inspection department**						
guidance						−0.503
						(−1.383,0.375)
**Performance appraisal**						
Once a month						0.199^a^
						(0.007,0.391)
2 times / month						0.052
						(−0.471,0.575)
3 times / month						0.289^a^
						(0.052,0.527)
4 times or more / month						0.423^a^
						(0.191,0.654)
**Publicity (frequency)**						
Once a year						0.514
						(−0.065,1.092)
2 times / year						1.073^a^
						(0.482,1.665)
3 times / year						0.764^a^
						(0.114,1.413)
4 times or more / year						0.909^a^
						(0.354,1.464)
**Training (frequency)**						
Once a year						0.837^a^
						(0.178,1.495)
2 times / year						0.526
						(−0.157,1.209)
3 times / year						1.008^a^
						(0.345,1.671)
4 times or more / year						0.729^a^
						(0.043,1.415)

^a^Significant at 0.05

## Discussion

To our knowledge, this is the first region-wide survey study to examine the management situation of submitting specimens to a microbiology laboratory for inspection before use of antimicrobial. We explored the influencing factors based on the perspective of management and focused on the effect in different level of management factors. This study described the management status of the departments involved in the process of PP, and found that different departments have different degrees and effects of management and support related to PP and there was a significant difference in the PP between secondary and tertiary hospitals.

### The single composition of the members of the infection management department affected the management effect during the pre-analytical process

From the organizational level, the antibacterial drug management plan was carried out by the infection management department in most hospitals. However, we found that the professional background of the organization team for infection management in the hospital is single, mainly nurses. Since a pre-prescription antibiotic inspection requires the cooperation of multiple departments, the management team should achieve a cross-professional combination to improve inspection efficiency and value. The diagnostic management plan proposes that laboratories working with the management team can reduce unnecessary tests and false positive results and better improve patient care [25]. Knobloch et al., suggested that we need to adjust the management plan in combination with the local need and influencing factors [26].

### Management factors such as guidance, training, publicity and performance appraisal have different impacts on each department

The antimicrobial stewardship program usually focuses on education interventions in many countries [27, 28]. Studies on improving the quality of hospital infection prevention and control have proposed to strengthen training, publicity and performance appraisal [26, 29]. However, our research found that different frequencies of training, performance appraisal and publicity have different effects on the quality of the pre-analytical process, and also have different effects in different departments.

For clinicians in key departments, although the request rates of different types of antimicrobial basically met the requirements, we found that there were differences between different levels of hospitals, and the impact of management factors on them was different. Different from the other two types of antimicrobial, only low-frequency performance appraisal has a significant impact on non restricted-use antimicrobial. Non restricted-use antimicrobial were usually provided in empirical drug use schemes, and the demand for microbiological test results is not very necessary, so the management requirements are relatively loose. This finding suggests that for most clinicians, an overly frequent review regime may be detrimental to the promotion of PP on non-restricted antimicrobial. Some studies have noted that employees’ perceptions of fairness in performance appraisal also impact employee performance, including perceptions of the rationality of the evaluation cycle. In addition, the rationality of performance evaluation content for different levels of employees also affects their performance [21]. We also found that the hospital’s publicity activities on clinicians did not have a positive impact, and even had a negative impact in collection department and transportation Department. It is understood that the PP publicity in the survey area is usually distributed in the form of brochures. This single form is mostly considered ineffective. For the specimen collection department, it can be seen that providing a standard operation guide and training is very important to reduce errors in the collection process, and our results were in line with previous studies [29]. At the same time, we are also concerned that the improvement of collection equipment and technology is also an urgent need to improve the quality of PP. It is suggested that hospital managers need to pay attention to what is the real management demand to improve the inspection work, and the technical and awareness intervention measures of different departments related to the inspection should be different in terms of methods and degrees [14]. For the transportation department, we found that due to a lack of clear regulations regarding the establishment and ownership of the department, many hospitals lack targeted supervision systems. The problems in specimen transport were not resolved in time, which became the most easily neglected link affecting the quality of PP. For the inspection department, we found that the provision of guidance, training and appraosals had a significant positive impact on PP. However the inspection department had fewer performance appraisals related to PP and few laboratory communicate with clinical or other departments during the PP. The pre- and post- analysis phases were considered to be the stage with high incidence of laboratory inspection errors and lack of attention. Researchers emphasized that it is necessary to improve the laboratory personnel’s attention to the sample quality in the PP and the interpretation and feedback of the results after analysis [24]. Therefore, it is necessary for managers to strengthen the appraisals of inspectors in the above process.

### Information technology can help to improve the management quality of the pre-analysis process

In addition, we found the positive impact of information technology on improving the quality of PP. Many studies have proposed that the first way to improve the nursing quality and safety of inpatients is to use a well-designed information system to prevent and manage laboratory errors, and promote the continuous exchange of information among doctors, nurses and laboratory experts [30, 31]. Our results are consistent with them. Compared with regular education and training, information system can provide more timely decision-making guidance. A study from Portugal showed that good communication between doctors and microbiology laboratories should be considered a priority for any ASP [11]. Efforts should be made to improve data sharing between doctors and microbiological laboratories, such as the provision of antibiotic prescription guidelines suitable for local hospital epidemiology, and easy access to hospital antibiotic sensitivity models and epidemiological databases. Multiple Department roles are designed in the inspection process. Good communication can reduce the risk of errors before laboratory analysis. As a timely tool, information technology can promote multi-party information sharing and communication. However, at present, in most of the tertiary and all secondary medical institutions, there is no perfect information system to support the inspection, and the functional requirements of the information system need to be further collected in various departments.

A limitation of the study was that the content of the survey was not comprehensive enough. This could have introduced some bias and omit some possible influencing factors, however the relatively professionals involved in the management of inspection request, providing advice from real practice for survey item, could have reduced this risk. The distribution of the survey may be another limitation. The number of departments surveyed was limited and may not be representative of the wider hospital in this country. Since our survey is the first baseline survey in China, the selected hospitals in a central province are enough to reflect the current average level of examination.

## Conclusion

This study describes the level of microbiological examination before the use of antimicrobial in hospitals in Central China, and discusses the influence of management factors. It is found that there are differences in the examination level between different levels of hospitals and obstacles in different departments. Management factors such as guidance, training, publicity and performance appraisal have different effects on different departments in different frequencies, which affects the quality of hospital examination to a certain extent. In addition, the information technology scheme helps to promote clinicians’ examination request and provides the possibility for timely communication among departments. Our research provides valuable evidence for further improving the inspection request work.

## Supplementary Information

**Additional file 1.** Questionnaire.

## Data Availability

The data used and analyzed during the current study are available from the corresponding author on reasonable request.
