# Supplementary material for: The pre-analytical process management status and influencing factors of laboratory test before prescribing antimicrobial in developing country
Source: BMC Health Serv Res. 2023 Mar 25;23:283. doi: 10.1186/s12913-023-09243-8 (PMC10039769; doi:10.1186/s12913-023-09243-8)
Supplement: Supplementary file 1 — Additional file 1. Questionnaire. [file 12913_2023_9243_MOESM1_ESM.docx]

Questionnaire

Part 1

Dear head of the hospital infection control department:

The improvement of the rate of pathogenic examination before the treatment of antibacterial drugs can effectively improve the scientific and normative use of antibacterial drugs, which is of great significance to the continuous improvement of hospital medical quality. In order to implement the 10 medical quality and safety improvement goals put forward by the National Health and Health Commission in 2021, and respond to the requirements of the Hubei Provincial Health and Health Commission in the corresponding "323" key action work to improve the rate of pathogenic examination of inpatients before the treatment of antibacterial drugs, it is necessary to understand the current working process and quality management of pathogenic examination in your hospital. Please fill in the following contents according to the actual situation of your hospital's etiological inspection work to provide a basis for the quality improvement evaluation in the later stage.

| Gender |  |
| --- | --- |
| Age |  |
| Professional background | Clinical medicine  Nursing  Public Health  Laboratory Science  Pharmacy |
| Education | Doctor  master  undergraduate  junior college  junior college or below |
| Title | Senior and senior associate  Intermediate  Primary and below |
| Working years | Year(s) |
| Section 2 Quality management and monitoring of hospital pathogenic examination |  |
| The hospital has a regulatory system or regulations for the submission of microbiological specimens for inspection before antimicrobial treatment (SMI) | Yes  No |
| The hospital has professionals or organizations responsible for the quality control of SMI. | Yes  No |
| There are corresponding quality control indicators | Yes  No |
| The hospital has the performance appraisal indicators of the relevant departments for the pathogen inspection before antimicrobial treatment | Yes  No |
| Frequency of performance appraisal | Never  Once a year  2 times/year  3 times/year  4times or more/year |
| There are corresponding quality control indicators (such as the rate of pathogenic examination before SMI, the rate of pathogenic examination before the treatment of restricted use of grade antibacterial drugs in inpatients, etc.) | Yes  No |
| Frequency of publicity and education activities or training related to the pathogen inspection organized by our hospital | Never  Once a year  2 times/year  3 times/year  4times or more/year |

Part 2 clinicians

Dear doctors

In response to the 10 medical quality and safety improvement goals proposed by the National Health and Health Commission in 2021, and the requirements of the Hubei Provincial Health and Health Commission in the corresponding "323" campaign to improve the rate of pathogenic examination of inpatients before the treatment of antibacterial drugs, it is now necessary to understand the current work situation of your hospital's pathogenic examination application. Please fill in the following contents according to the actual situation of your hospital's etiological inspection work to provide a basis for the quality improvement evaluation in the later stage.

| Gender |  |
| --- | --- |
| Age |  |
| Department |  |
| Professional background | Clinical medicine  Nursing  Public Health  Laboratory Science  Pharmacy |
| Title | Senior and senior associate  Intermediate  Primary and below |
| Working years | Year(s) |
| You will submit the application of microbiological specimens for inspection before non-restricted antibiotics treatment | Yes  No  Not sure |
| You will submit the application of microbiological specimens for inspection before restricted antibiotics treatment | Yes  No  Not sure |
| You will submit the application of microbiological specimens for inspection before special grade antibiotics treatment | Yes  No  Not sure |
| How often do you participate in the relevant training of SMI? | Never  Once a year  2 times/year  3 times/year  4times or more/year |
| How often do you participate in the publicity activities related to SMI? | Never  Once a year  2 times/year  3 times/year  4times or more/yea |
| The hospital has formulated the guideline of the standard operating procedures for the application of SMI. | Yes  No |
| The frequency of performance appraisal for SMI. | Never  Once a month  2 times/month  3 times/month  4 times or more/month |
| There is an information system for sharing the inspection data. | Yes  No |
| There is process monitoring and early warning for relevant data, such as critical value report. | Yes  No |
| Pathogenic test is the prerequisite for prescription of antibacterial drugs in information system. | Yes  No |
| After obtaining the results of pathogenic test, you will receive a prompt setting to adjust the treatment plan of antibacterial drugs. | Yes  No |
| Whether the hospital has carried out the following pathogenic test items (tick "Yes"). | ⏵ Bacterial culture  ⏵ Fungal culture  ⏵ Calcitonin detection  ⏵ Interleukin-6 detection  ⏵ G test |
| Notes: The respondents required that there should be at least 5 clinicians in each department of respiratory medicine, urology, ICU (severe, respiratory and severe, children's severe), neurology, endocrinology and orthopedics who have the right to prescribe antibiotics. If the doctors of each department do not meet the quantity requirements, investigate all doctors of the corresponding department; If the hospital has only general practice/major internal medicine/major surgery, all doctors in the hospital should be investigated. | |

Part 3 nurses

Dear nurses

In response to the 10 medical quality and safety improvement goals proposed by the National Health and Health Commission in 2021, and the requirements of the Hubei Provincial Health and Health Commission in the corresponding "323" campaign to improve the rate of pathogenic examination of inpatients before the treatment of antibacterial drugs, it is necessary to understand the current work of sample collection in the pathogenic examination of your hospital. Please fill in the following contents according to the actual situation of your hospital's etiological inspection work to provide a basis for the quality improvement evaluation in the later stage.

| Gender |  |
| --- | --- |
| Age |  |
| Department |  |
| Professional background | Clinical medicine  Nursing  Public Health  Laboratory Science  Pharmacy |
| Title | Senior and senior associate  Intermediate  Primary and below |
| Working years | Year(s) |
| The hospital has developed a laboratory guide for the collection of samples for SMI, and the guidance manual of this service guide has been distributed to the medical wards where the department provides samples. | Yes  No |
| Relevant records after collection of pathogenic samples | Yes  No |
| When you collect samples, you encounter the following problems that will affect the quality of samples | ⏵ Contamination of the collection site's own flora. () times/week  ⏵ Incorrect selection of the collection site. () times/week  ⏵Lack of appropriate technology and equipment to collect samples. () times/week ⏵Insufficient collection of samples. () times/week  ⏵No labeling of patients and collection related information. () times/week  ⏵Lack of appropriate sample placement containers. () times/week |
| How often do you participate in the relevant training of SMI? | Never  Once a year  2 times/year  3 times/year  4times or more/year |
| How often do you participate in the publicity activities related to SMI? | Never  Once a year  2 times/year  3 times/year  4 times or more/year |
| There is a performance appraisal system for SMI in our department. | Yes  No |
| The frequency of performance appraisal for SMI. | Never  Once a month  2 times/month  3 times/month  4 times or more/month |
| Notes: The respondents required that at least 8 nurses from each department of respiratory medicine, urology, ICU (severe, respiratory and severe, children's severe), neurology, endocrinology and orthopedics should participate in the collection of pathogens. If the number of nurses in each department does not meet the requirements, investigate all the nurses in the corresponding department; If the hospital has only general practice/major internal medicine/major surgery, all nurses in the hospital should be investigated. | |

Part 4 specimen transporters

Dear specimen transporters

In response to the 10 medical quality and safety improvement goals proposed by the National Health and Health Commission in 2021, and the requirements of the Hubei Provincial Health and Health Commission in the corresponding "323" campaign to improve the rate of pathogenic examination of inpatients before treatment of antibacterial drugs, it is necessary to understand the current work of sample delivery in your hospital's pathogenic examination. Please fill in the following contents according to the actual situation of your hospital's etiological inspection work to provide basis for the quality improvement evaluation in the later stage.

| Gender |  |
| --- | --- |
| Age |  |
| Department |  |
| Professional background | Clinical medicine  Nursing  Public Health  Laboratory Science  Pharmacy |
| Title | Senior and senior associate  Intermediate  Primary and below |
| Working years | Year(s) |
| The hospital has developed a laboratory service guide for the operation procedures for the delivery of pathogenic samples for SMI, and the guidance manual of this service guide has been distributed to the medical wards where the department provides samples. | Yes  No |
| Are there any interference factors that affect the time and quality of samples during the inspection? | Yes  No |
| Possible interference factors include | ⏵Too many samples, too late to be sent for inspection  ⏵Storage conditions are harsh, there is no suitable container  ⏵the distance is too long, the transit time is too long  ⏵the packaging and marking of the samples are not standardized, and need to be modified  ⏵Not handled in time due to insufficient inspection personnel |
| How often do you participate in the relevant training of SMI? | Never  Once a year  2 times/year  3 times/year  4times or more/year |
| How often do you participate in the publicity activities related to SMI? | Never  Once a year  2 times/year  3 times/year  4 times or more/year |
| The frequency of performance appraisal for SMI. | Never  Once a month  2 times/month  3 times/month  4 times or more/month |
| Notes: The respondents required: at least 2 clinical examination personnel from each department of respiratory medicine, urology, ICU (severe, respiratory and severe, child severe), neurology, endocrinology and orthopedics. If the number of clinical inspectors in each department does not meet the requirements, investigate all clinical inspectors in the corresponding department; If the hospital has only general department/major internal medicine department/major surgery department, then investigate all clinical examination personnel of the hospital. If the medical institution is sent for examination by the nurse, it shall be filled in by the nurse who is responsible for sending for examination in each department. | |

Part 5 specimen transporters

Dear specimen transporters

In response to the 10 medical quality and safety improvement goals proposed by the National Health and Health Commission in 2021, and the requirements of the Hubei Provincial Health and Health Commission in the corresponding "323" campaign to improve the rate of pathogenic examination of inpatients before treatment of antibacterial drugs, it is necessary to understand the current work of sample delivery in your hospital's pathogenic examination. Please fill in the following contents according to the actual situation of your hospital's etiological inspection work to provide basis for the quality improvement evaluation in the later stage.

| Gender |  |
| --- | --- |
| Age |  |
| Department |  |
| Professional background | Clinical medicine  Nursing  Public Health  Laboratory Science  Pharmacy |
| Title | Senior and senior associate  Intermediate  Primary and below |
| Working years | Year(s) |
| The laboratory has a clear quality policy and quality objectives for the pathogenic test before the treatment of antibacterial drugs (such as the timely rate of report distribution and the timely rate of critical value report) | Yes  No |
| The laboratory has process guidelines or rules and regulations for the pathogenic test work | Yes  No |
| For unqualified samples, you can communicate with clinicians or nurses in time | Yes  No |
| Hold joint meetings with clinical departments on a regular or irregular basis to discuss issues related to sample collection, transportation, inspection and report of etiological examination | Never  Once a year  2 times/year  3 times/year  4times or more/year |
| How often do you participate in the relevant training of SMI? | Never  Once a year  2 times/year  3 times/year  4times or more/year |
| How often do you participate in the publicity activities related to SMI? | Never  Once a year  2 times/year  3 times/year  4 times or more/year |
| The frequency of performance appraisal for SMI. | Never  Once a month  2 times/month  3 times/month  4 times or more/month |
| Notes: Inspectors of all medical institutions (at least 2 in the secondary hospitals and at least 3 in the tertiary hospitals). If the inspection personnel of each institution do not meet the quantity requirements, all the inspection personnel of the investigation institution | |
